# 93 Burn Patient Perspectives on Disability Weights and the Philosophy of Disability

**DOI:** 10.1093/jbcr/irae036.092

**Published:** 2024-04-17

**Authors:** Paul Won, Karel-Bart Celie, Haig A Yenikomshian

**Affiliations:** Keck School of Medicine, Los Angeles, CA; University of Southern California, Los Angeles, California; Keck School of Medicine, Los Angeles, CA; University of Southern California, Los Angeles, California; Keck School of Medicine, Los Angeles, CA; University of Southern California, Los Angeles, California

## Abstract

**Introduction:**

Disability-adjusted life years (DALYs) have a ubiquitous presence in academic global health, including attempts to understand the global burden of burn injuries. An important component of the DALY is the “disability weight” (DW)—a number from 0 to 1 that assigns a “badness” to the disease state in question. The numbers assigned by DWs depend on the individuals being queried, as well as the underlying philosophical model of disability that is being assumed. The present scoping review aimed to examine: first, whether DWs for burns were derived from actual burn patients (rather than from clinicians/lay public); second, whether the existing literature indicates which of the three most common philosophical models of disability aligns with burn patient experiences.

**Methods:**

A systematic search of six databases was conducted for articles published any time until January of 2023. Inclusion criteria were studies in English, studies with human subjects, and studies investigating burn patient perspectives on disability. Study aims, methodology, and results were collected, and general themes were identified and described. The Critical Appraisal Skills Program (CASP) checklist was utilized to evaluate included studies.

**Results:**

764 articles were returned, and 10 met criteria for in-depth review. Regarding our first aim, no studies were found that directly queried burn patient for DWs. Regarding our second aim, 4 studies contained data that could be extrapolated to burn patient perspectives on the philosophy of disability. These articles utilized semi-structured interviews and reported qualitative analysis of thematic elements such as return to work and social integration. In all studies, a recurring theme was that burn injuries are instrumentally detrimental, with modulation based on the patient’s social environment. For example, patients with manual occupations often reported a significant burden of disease and cited resumption of work as a significant factor to their social adjustment and recovery. This aligns most with a philosophical model of disability known as the welfare account.

**Conclusions:**

This scoping review highlights a significant gap in literature. First, no studies were found that directly queried burn patients for DWs. Second, the philosophical model informing the concept of ‘disability’ in global health remains under-explored. These two themes should be prospectively studied in the burn population.

**Applicability of Research to Practice:**

Currently employed DWs use public and health expert opinions with limited input from burn patients. This study demonstrates two significant gaps in the literature that emphasize the importance of obtaining burn patient perspectives. A solid foundation for how ‘disability’ is understood—and subsequently employed in DWs—will help to more accurately triage resources and improve patient care.

